# Precursor sticking coefficient determination from indented deposits fabricated by electron beam induced deposition

**DOI:** 10.3762/bjnano.16.4

**Published:** 2025-01-13

**Authors:** Alexander Kuprava, Michael Huth

**Affiliations:** 1 Physics Institute, Goethe University Frankfurt, Max-von-Laue-Str. 1, 60438 Frankfurt am Main, Germany

**Keywords:** adsorption, continuum model, FEBID, nanofabrication, sticking coefficient

## Abstract

A fast simulation approach for focused electron beam induced deposition (FEBID) numerically solves the diffusion–reaction equation (continuum model) of the precursor surface on the growing nanostructure in conjunction with a Monte Carlo simulation for electron transport in the growing deposit. An important requirement in this regard is to have access to a methodology that can be used to systematically determine the values for the set of precursor parameters needed for this model. In this work we introduce such a method to derive the precursor sticking coefficient as one member of the precursor parameter set. The method is based on the analysis of the different growth regimes in FEBID, in particular the diffusion-enhanced growth regime in the center region of an intentionally defocused electron beam. We employ the method to determine the precursor sticking coefficient for bis(benzene)chromium, Cr(C_6_H_6_)_2_, and trimethyl(methylcyclopentadienyl)platinum(IV), Me_3_CpPtMe, and find a value of about 10^−2^ for both precursors, which is substantially smaller than the sticking coefficients previously assumed for Me_3_CpPtMe (1.0). Furthermore, depositions performed at different substrate temperatures indicate a temperature dependence of the sticking coefficient.

## Introduction

Nanoscale fabrication of free-form structures via methods like focused electron or ion beam induced deposition (FEBID/FIBID) requires precise beam control and sufficient knowledge of key properties of the precursor material used [1]. In addition, a reliable prediction of the expected deposit shape is needed and can be obtained based on a simulation of the FEBID process using the so-called continuum model that can be of great assistance for the nanofabrication process optimization [2–3]. Here again, sufficiently accurate knowledge of the values for the model-dependent set of precursor parameters is key in order to obtain correct results. In turn, this indicates that FEBID deposit shape analysis in conjunction with simulations may provide a suitable approach to identify the required values for the precursor parameters.

One of these precursor parameters is the sticking coefficient *s*. It represents the probability of a volatile precursor molecule to adhere or stick to the surface it impacts. The coefficient takes values from 0 to 1, where at 0 none of the incoming molecules stick to the surface and at unity all of them do. It accounts for events of prompt scattering of impinging molecules on the free surface sites. Such an event can be pictured as an interaction where no van der Waals “bond” is established and where the molecule leaves the surface at a time scale much shorter than the residence time τ [4]. In the continuum model, *s* is one of the model parameters entering the diffusion–reaction equation (RDE) in the adsorption term, where the total local precursor flux is multiplied by the sticking coefficient to characterize the supply of precursor molecules to the surface [5].

In the context of FEBID, the sticking coefficient has not received much attention; only in one work it was deliberately determined [6]. In some other works, the sticking coefficient has been used in an expression describing the value of precursor supply frequency which was sufficient for the investigated case [7–8]. A number of works using the continuum model assumed the sticking coefficient to be unity as a rough estimate or initial guess in calculations or even simulations dedicated to FEBID for precursors such as WF_6_ [9], Me_3_CpPtMe [10–15], HCo_3_Fe(CO)_12_, and Nb(NMe_2_)_3_(N-*t*-Bu) [12], as well as tetraethyl orthosilicate (TEOS) [16]. The sticking coefficient has been determined only in the work of Fowlkes and Rack [6] where a value of 0.025 was reported for W(CO)_6_. In this work, a stationary pulsed beam was used to study the adsorption/desorption dynamics. A fit of the results to the continuum model was performed with an estimated value for the energy-integrated dissociation cross section in order to obtain values for the diffusion coefficient (*D*), residence time (τ), and sticking coefficient (*s*). In this case, the sticking coefficient corresponds to “precursor-to-deposit” sticking rather than to “precursor-to-substrate” sticking as the actual substrate for continued growth is the deposit itself once the first closed layer is formed.

In another series of surface-science-oriented works, the sticking coefficient has been studied for small organic molecules, such as allyl methyl ether on Si(100) [17], trimethylamine on Si(001) [18], tetrahydrofuran on Si(001) [19], and benzene on Pt(111) [20]. A molecular beam gun equipped with a shutter was used in conjunction with mass spectrometry. The sticking coefficient was deduced from the measured drop in the spectrometer signal upon opening the shutter. In [17,19] the sticking coefficient was determined at different substrate temperatures and a transitional behavior was found when going from about 100 K to 1000 K. At 100 K the sticking coefficient was found to be close to unity and it dropped to near zero closer to 1000 K. All the aforementioned works report precursor-mediated adsorption dynamics, whereby molecules transiently adsorb after surface saturation with an initial layer of adsorbate. Effectively, this means that the obtained sticking coefficients refer to “precursor-to-precursor” sticking rather than to “precursor-to-substrate” sticking. The investigations were also done for different kinetics energies of the impinging molecules revealing a decrease of the sticking coefficient with increasing energy. In comparison, the molecules investigated in all of these studies are significantly smaller than the organometallic precursors typically used in FEBID. Nevertheless, these findings are important in order to understand the adsorption kinetics of larger molecules as used in FEBID.

In the work presented here, we introduce a method to determine the value for the “precursor-to-deposit” sticking coefficient of precursors used in FEBID based on the continuum model. The method implies a steady-state FEBID growth regime that allows to obtain an indent-shaped deposit using a stationary and significantly defocused beam. We show that the continuum model and conclusions from it allow to obtain sticking coefficient values when using realistic estimates of local surface precursor flux and dissociated molecule fragment volume. Additionally, we show that it is possible to obtain the sticking coefficient value without the indented deposit if additional precursor parameters are known with sufficient accuracy.

### The model

At the heart of the continuum model is the reaction–diffusion equation that describes the local precursor surface concentration or site density *n*. This concentration is controlled by four processes: adsorption, desorption, dissociation, and diffusion. Here, we formulate the equation under radially symmetric process conditions:


[1] $$\frac{\partial n}{\partial t}=s\Phi \left(1-\frac{n}{n_{0}}\right)-\frac{n}{\tau }-\sigma fn+D\left(\frac{\partial^{2}n}{\partial r^{2}}+\frac{1}{r}\frac{\partial n}{\partial r}\right),$$

where *s* is the sticking coefficient, Φ is the precursor flux at the surface, *n*_0_ is the maximum precursor site density, τ is the average precursor residence time, σ is the energy-averaged dissociation cross section, and *D* is the surface diffusion coefficient. This rate equation makes up the balance between all processes that contribute to replenishment and depletion of precursor molecules.

The electron beam is described by a Gaussian shape function:


[2] $$f\left(r\right)=f_{0}\exp\left(-\frac{r^{2}}{2a^{2}}\right),$$

where *f*_0_ is the maximum electron flux at *r* = 0 and *a* is the standard deviation. The width of the Gaussian is defined as 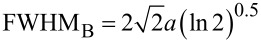.

The growth rate under electron irradiation is proportional to the local electron flux, the dissociation cross section, and the volume *V* of the deposited non-volatile fragment. Under stationary steady-state deposition conditions, the growth rate can be expressed as:


[3] R(r)=n(r)σ f(r)V.

Under such conditions the final shape of the deposit is a product of the radially resolved growth rate and the deposition time.

In the following we use a dimensionless growth rate which is independent of the deposited molecule fragment volume *V* and impinging precursor flux [7]:


[4] $$\bar{R}=\frac{R}{s\Phi V}.$$

This normalized growth rate allows us to abstract away from the process details and look at it from a more general perspective.

**Figure 1:**
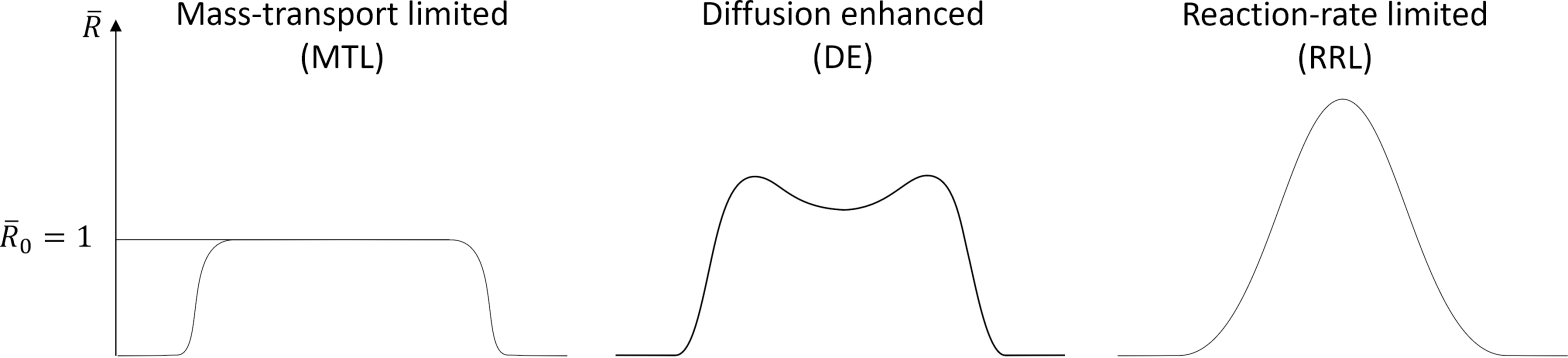
Deposit shapes from the three distinct growth regimes.

Depending on the deposition conditions and employing a Gaussian beam, three distinct deposit shapes evolve in accordance with the deposition regime (Figure 1) as governed by the balance between the four processes in Equation 1. The first case with a flat-top deposit is typical for the precursor- or mass-transport-limited regime (MTL). In this case, precursor replenishment by diffusion into the beam-impact region (BIR) is close to zero, meaning that diffusing precursor molecules are dissociated before entering the area roughly defined by the full width at half maximum (FWHM) of the beam border. In contradistinction, a Gaussian-like deposit shape (rightmost shape) is typical for the electron- or reaction rate-limited regime (RRL) when diffusive replenishment is strong enough to supply the precursor to the center of the BIR. The stronger the diffusion, the closer the shape is to a Gaussian. The shape with an indent at the center emerges for the intermediate case (i.e., in the diffusion-enhanced (DE) regime). It should be noted that these regimes are assumed to emerge during steady-state growth.

Considering now the MTL regime, the growth rate from Equation 3 at the beam center can be rewritten for the case without diffusion and under stationary conditions for Equation 1 as:


[5] $$R=s\Phi \tau_{\text{in}}\sigma \;f_{0}V,$$

where


[6] $$\tau_{\text{in}}={\left(\frac{s\Phi }{n_{0}}+\frac{1}{\tau }+\sigma \;f_{0}\right)}^{-1}.$$

Then, with the Equation 4, the dimensionless growth rate is:


[7] $$\bar{R}=\tau_{\text{in}}\sigma \;f_{0}.$$

Substituting τ_in_ from Equation 6 we obtain:


[8] $$\bar{R}=\frac{\sigma \;f_{0}}{\frac{s\Phi }{n_{0}}+\frac{1}{\tau }+\sigma \;f_{0}}.$$

In the case of significant dissociative depletion 
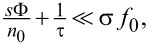
 Equation 8 yields 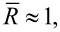 which is a key feature of the extreme MTL regime and corresponds to the growth rate on the flat-top deposit in Figure 1.

This can also be applied to indented deposits under certain conditions. Indented deposits are typically formed when using a precursor with a larger surface diffusion coefficient and a wide large-diameter beam, for example. The appearance of an indent or, for wider deposits, a brim around a central plateau region has two conditions: 1) The beam current has to be high enough to cause significant depletion and thus induce diffusion. Low irradiative depletion would shift the balance towards the RRL regime. 2) The precursor has to exhibit a certain degree of diffusivity in order to guarantee a noticeable mass transport.

**Figure 2:**
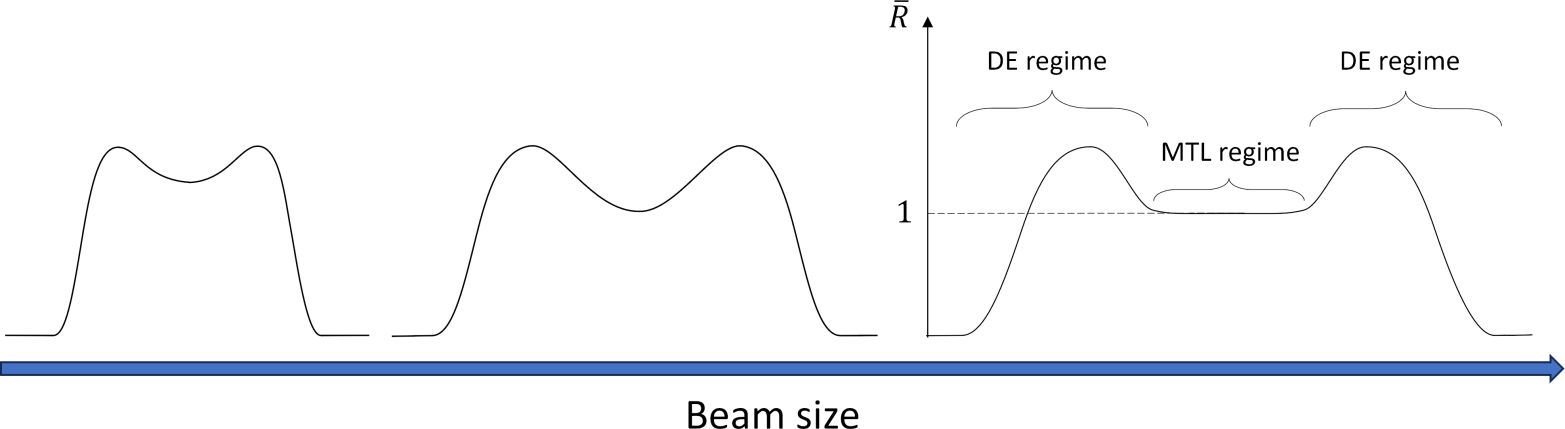
Evolution of an indented deposit with beam size and growth regimes of an indented deposit with a plateau.

The depth of the indent can be controlled by the degree of the beam defocus, although there is a lower limit of the indent depth. With a significantly wide beam, the indent reaches its maximum depth and evolves into a plateau (Figure 2). In such a case, all diffusing precursor molecules are consumed long before reaching the center of the BIR. Thus, a large central part of the deposit is completely cut off from diffusive replenishment, which leads to the emergence of an extreme MTL regime surrounded by a DE regime.

The diffusion contribution at the center of the beam can be neglected under certain conditions. The beam size has to be large enough for all mass transport to be consumed by the beam before it reaches the beam center area. To fulfill this condition in practice, the beam size and current have to be chosen such that a well-defined plateau shape is formed. This precaution is taken as it has been previously shown that diffusion may contribute to precursor replenishment at the center of the beam even in a seemingly strong MTL regime [21]. A more detailed discussion is found in Supporting Information File 1, Section 3.

As the central region conforms to the MTL regime, 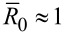 is true for a wide flat central part of the deposit. The condition 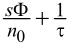 ≪ σ*f*_0_ proves true for typical precursor dissociation cross sections and higher beam currents that are ultimately needed for the method described here. By using 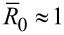 together with a sufficiently reliable estimation of the surface precursor flux Φ and the volume deposited from a single precursor molecule *V* in Equation 4, the sticking coefficient *s* can be determined from


[9] $$s=\frac{R_{0}}{{\bar{R}}_{0}\Phi V},$$

where *R*_0_ is the growth rate at the center of the deposit (i.e., in the plateau region where 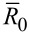 is equal to unity).

## Results

Two precursors were used to test the applicability of the method to quantify the sticking coefficient: bis(benzene)chromium, Cr(C_6_H_6_)_2_, and trimethyl(methylcyclopentadienyl)platinum(IV), Me_3_CpPtMe.

Initially, the precursor flux at the BIR was calculated from the total precursor flux estimated from the operating pressure during deposition, turbo pump pumping speed, and gas injection system (GIS) geometry using the GIS nozzle gas dynamics simulation approach described in [22]. The tilt of the GIS in relation to the substrate surface that defines the angle at which molecules hit the surface was 13° for Cr(C_6_H_6_)_2_ and 50° for Me_3_CpPtMe. Both GIS needles were 100 μm above the surface and 100 μm away from the beam axis. The base pressure of the instrument was 4 × 10^−7^ mbar, the chamber pressure during deposition which was used for the estimation of precursor flux was 5 × 10^−7^ mbar for Cr(C_6_H_6_)_2_ and 6 × 10^−6^ mbar for Me_3_CpPtMe, respectively. The precursor flux Φ for these precursors was thus estimated at 270 and 1900 nm^−2^·s^−1^, respectively. The volume deposited from a single precursor molecule *V* was assumed to be 0.2 nm^3^ for both precursors as a rough estimate with regard to these works [11–12,23]. Detailed estimation approaches for *V* and Φ can be found in Supporting Information File 1, Section 1 and 2 correspondingly.

The deposition time for each deposition was chosen considering two factors. On the one hand, the deposition shall occur under stationary steady-state conditions for the application of the described continuum model and growth regime assumptions. This favors shorter deposition times and consequently results in a height decrease. On the other hand, to reliably resolve deposit profile and reduce measurement errors, the height should not be too small. The chosen exposure time for each deposit was eventually a compromise between these two conflicting conditions.

**Figure 3:**
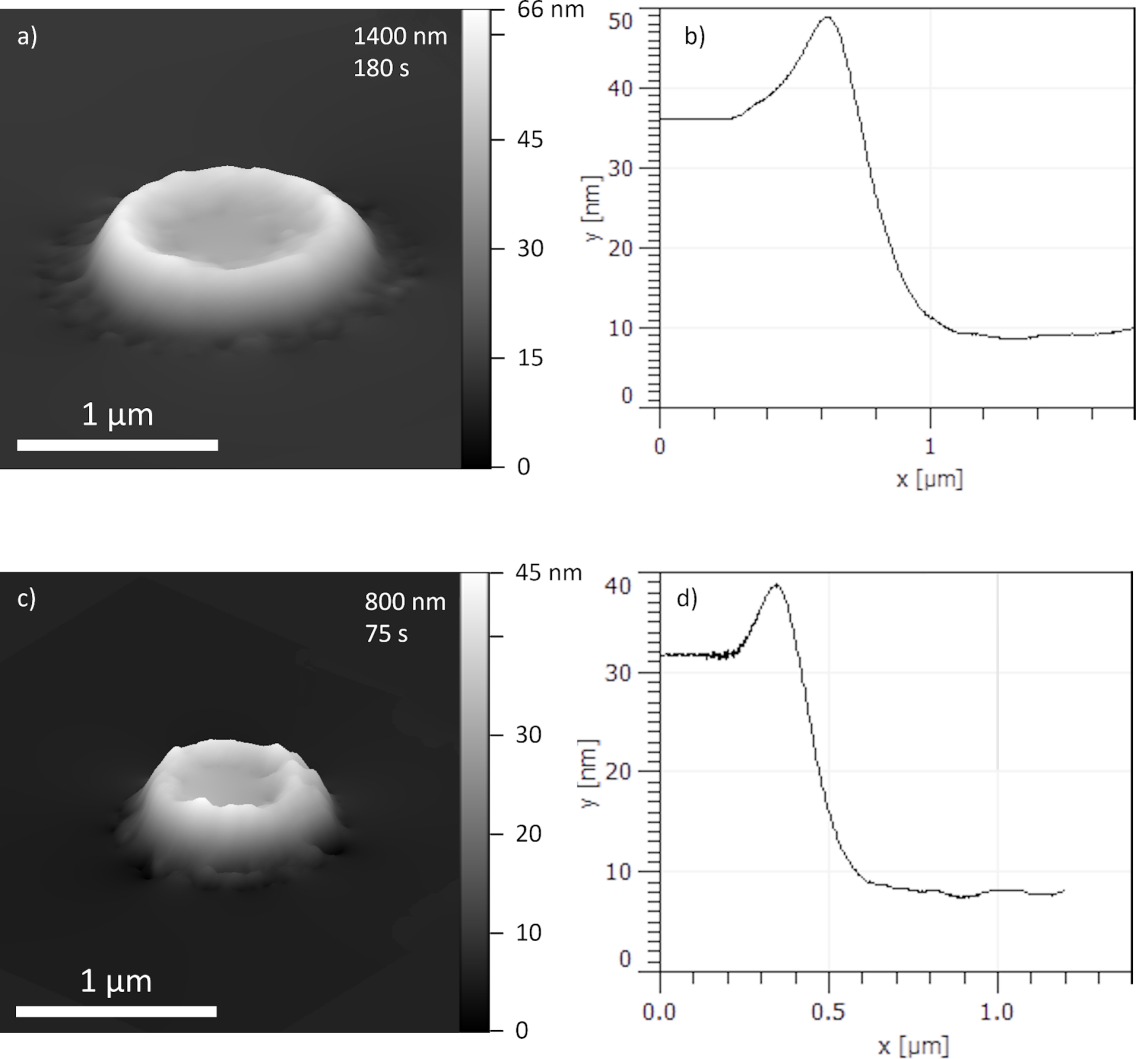
3D images from AFM scans of depositions with the Cr-precursor fabricated using 5 keV at a 400 pA beam (a, c). In the right corner are the beam size and deposition time. The corresponding radially averaged profiles are on the right (b, d). A small, blurred area at the plateau was averaged to obtain the central height. Significant beam defocus is required to produce a pronounced plateau at the deposit center.

Two deposits at a substrate temperature of 293 K were fabricated using Cr(C_6_H_6_)_2_ with different beam defocus setting. The AFM images of the deposits fabricated with 1400 and 800 nm wide beams (Figure 3) clearly exhibit an indent resembling a volcano. The size of 800 nm was the lowest possible beam size that produced a distinguishable plateau, since for smaller beam sizes the plateau disappears as diffusive replenishment starts reaching the BIR center and the DE regime prevails. Corresponding central growth rates are 0.15 and 0.25 nm/s, respectively. In comparison to the most commonly used precursors for FEBID, the Cr-precursor exhibits a rather low growth rate. The resulting sticking coefficients for these deposits are 0.0051 ± 0.0018 and 0.0090 ± 0.0038, respectively.

**Figure 4:**
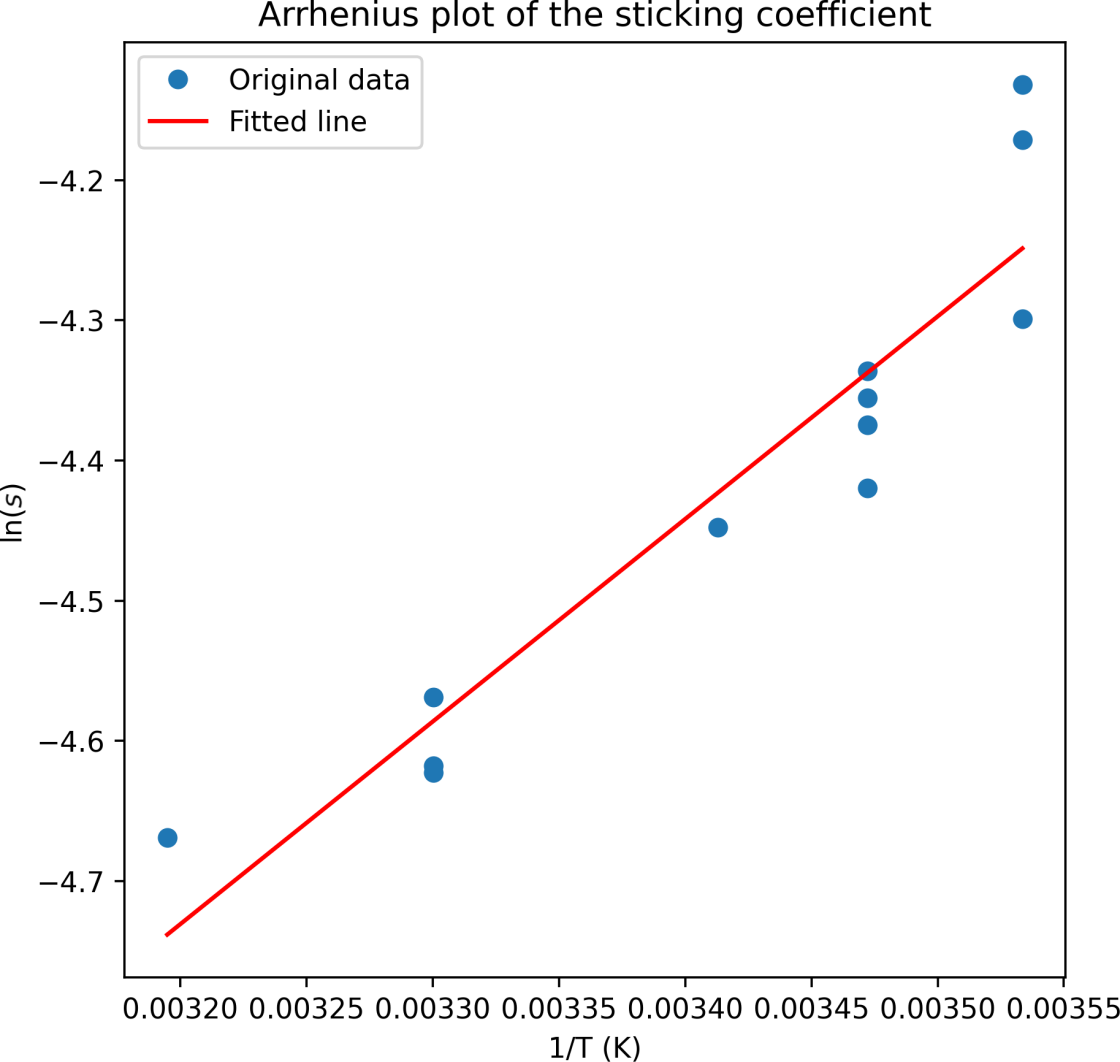
Sticking coefficients from deposits fabricated at different temperatures. Several deposits with varying deposition times were prepared at the same temperature. The red line is a fit over averaged values for each temperature.

Depositions with Cr(C_6_H_6_)_2_ have then been performed at different substrate temperatures and deposition times for the 800 nm beam size. The sticking coefficients were calculated using the method described above. The data in Figure 4 shows an Arrhenius plot of the calculated sticking coefficient.

**Figure 5:**
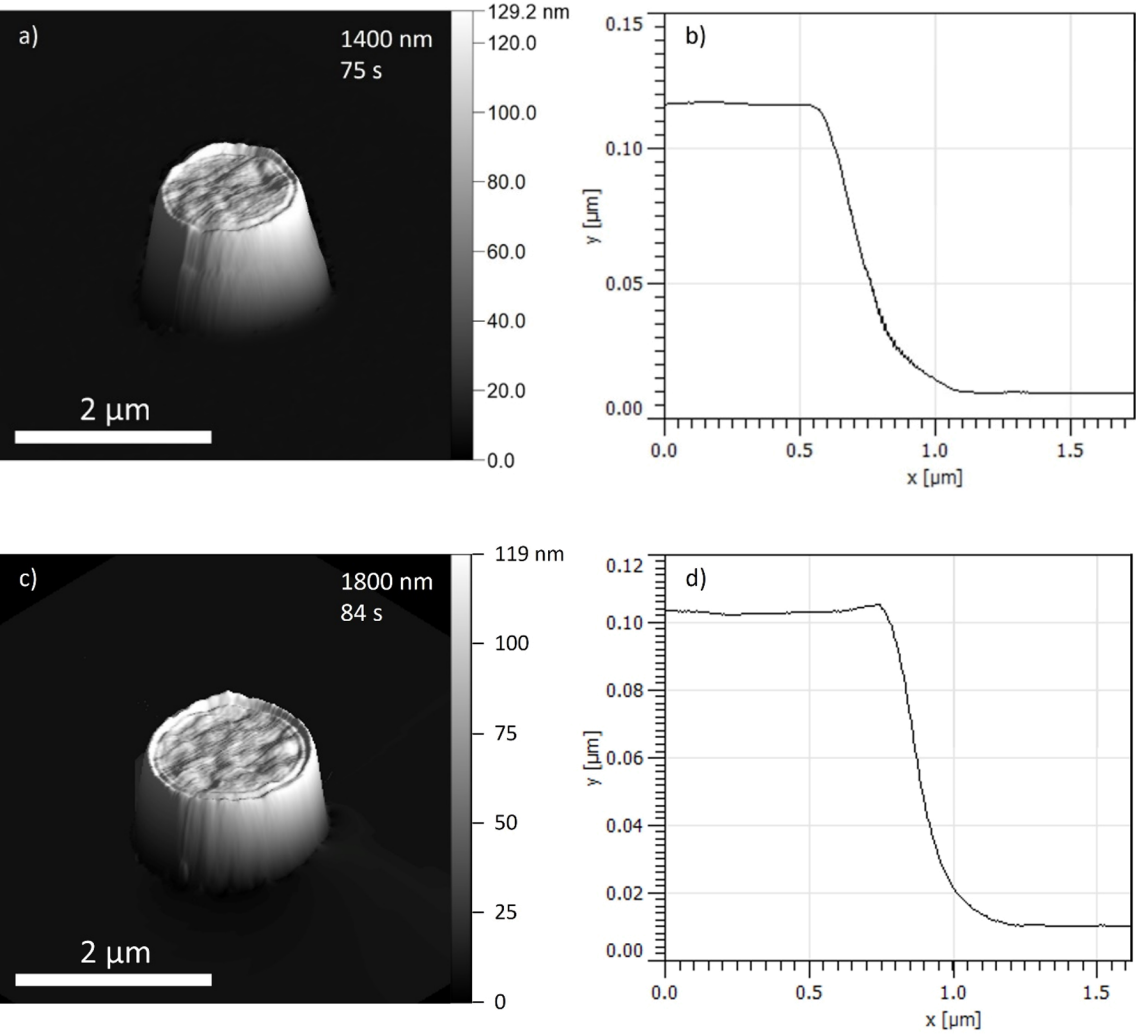
3D images from AFM scans of PtC deposits with different beam sizes (a, c) and radially averaged profiles (b, d). The brim at the edges correspond to a diffraction pattern rather than to an indent.

In addition, depositions have been performed with the widely used precursor Me_3_CpPtMe (PtC) at room temperature. The AFM scans in Figure 5 clearly indicate a flat-top deposit from which a growth rate of 1.75 nm/s can be deduced. In contradistinction to the volcano-shaped deposits observed for the Cr-precursor, with the Pt-precursor no elevated rim region develops, the reasons for which are discussed next.

## Discussion

The appearance of an indent in the deposits is attributed to a larger diffusion coefficient and a longer residence time which is required to induce a sufficient concentration gradient between the BIR and the outer regions under controlled irradiation conditions. In contrast to the Cr(C_6_H_6_)_2_ precursor, deposits using Me_3_CpPtMe did not show any indent even at the highest currents available for our instrument, which suggests that the diffusion coefficient and residence time for the Cr(C_6_H_6_)_2_ precursor are noticeably larger than the ones for Me_3_CpPtMe. The larger growth rate of the Pt-precursor is attributed to a larger precursor supply from the gas phase.

Although the use of the Pt-precursor did not result in a volcano-shape deposit, an estimation of the sticking coefficient for it is nevertheless possible with precursor parameter values previously assessed. Unlike the Cr(C_6_H_6_)_2_ precursor, which has not been studied within the present context before, Me_3_CpPtMe has been thoroughly investigated and the values for most of the key parameters are known, which enables the determination of the sticking coefficient without having a volcano-shaped deposit. Firstly, in the absence of a volcano shape, the growth regime has to be assessed. Using the parameters reported in [10–11], an estimation of the secondary electron (SE) surface flux of 5.4 × 10^5^ nm^−2^·s^−1^ at the BIR center with a 1400 nm wide beam at 5 keV and 400 nA on a gold-covered substrate and precursor flux estimation of 1900 nm^−2^·s^−1^, the dimensionless growth rate from Equation 8 equals to 0.71. This indicates a strong MTL regime. From here, using the height of the deposit fabricated under these conditions (see Figure 5a,b) and an estimated *V* = 0.2 nm^3^, a sticking coefficient of about 0.0057 ± 0.0025 can be derived. A very similar value is obtained for the wider deposit shown in Figure 5c,d.

The sticking coefficient values obtained for the Cr(C_6_H_6_)_2_ precursor are noticeably lower than the one reported for the W(CO)_6_ precursor (0.025), although the precursor flux and dissociated precursor molecule fragment volume are comparable. However, the value estimated for the Me_3_CpPtMe precursor is substantially lower than the estimate of 1 used in numerous other works mentioned in the introduction section.

The observed small difference between the sticking coefficients for Cr(C_6_H_6_)_2_ for two different beam sizes is within the error margins. Nevertheless, the cause could also be the presence of a process that influences precursor coverage but is not considered in the model. In this case, the difference is revealed by slightly different deposition conditions (current density). In particular, the model does not take into account the possibility of electron stimulated desorption (ESD) [24] which does depend on the current density and may negatively affect the precursor coverage in the plateau region of the deposit. In such a case, the sticking coefficient obtained from our method would be overestimated, the more so the higher the beam current would be. Thus, a possible alternative cause of the difference (0.0057 vs 0.009) could potentially be a higher ESD rate for the 800 nm beam as a consequence of a higher current density. It must be noted that the deposits fabricated with Me_3_CpPtMe do not show such a behavior. This could then be attributed to a significantly lower ESD influence for this precursor.

Another precursor behavior that may give a hint about the precursor stickiness is the pressure decay upon closing of the GIS. However, pressure decay can have several impacting factors rather than the calculated sticking coefficient itself. Firstly, the sticking coefficient of the molecules stemming from directed and diffuse flow will be different due to the different kinetic energies, as precursor gas from the gas injection system and precursor gas from surrounding surfaces are at different equilibrium temperatures. In other work involving lighter organic molecules [17], the sticking coefficient was shown to be inversely proportional to the kinetic energy of the molecule. Thus, the sticking coefficient for diffuse flux would be higher. Secondly, the longer molecules stay on the surface, the longer they remain in the chamber. This is reflected in the average residence time within the continuum model and not by the sticking coefficient which is the result of a transient state before the actual physisorption. Finally, the derived sticking coefficient is only valid for the “precursor-to-deposit” scenario. The values for both, sticking coefficient and residence time, may be different for metallic surfaces such as that of the chamber wall and internal installations. The aforementioned considerations can partially explain the substantially lower sticking coefficients determined here for FEBID-precursors than those determined for lighter organic molecules [17–20].

Finally, we address the observed slight temperature dependence of the sticking coefficient (see Figure 4). The sticking coefficient describes the probability of a molecule to adsorb to the surface, thus it depends on the adsorption activation energy. Assuming an Arrhenius-type behavior, an activation energy value *E*_a_ = 1.13 eV can be derived from the linear fit in Figure 4. The derived value is too big if physisorption is assumed, and it is two times bigger than the adsorption activation energy previously determined for Me_3_CpPtMe [11,13]. However, this derived value can be an overestimate of the true activation energy for physisorption considering a possible sticking coefficient overestimation. Thus, the value may well point towards physisorption, but a dedicated physisorption study would be needed to get a more accurate number. A dependency of the sticking coefficient on temperature has already been observed for lighter organic molecules [17,19–20]. The values in these works are close to unity at room temperature and lower temperatures and go down to near zero only at substantially high temperatures (>1000 K). In contradistinction, the values obtained for Cr(C_6_H_6_)_2_ at room temperature are almost two orders of magnitude lower. Despite the fact that the investigations in these works have been performed for relatively light molecules (≈72 g/mole) compared to Cr(C_6_H_6_)_2_ (208 g/mole) or Me_3_CpPtMe (319 g/mole), these findings may shed light on the adsorption dynamics of larger precursor molecules.

## Methods

A dual-beam microscope Nova 600 (FEI Company, the Netherlands) at Goethe University Frankfurt was used for the nanofabrication process. Structures were deposited on a 500 nm thick Au surface on top of a SiO_2_-terminated Si substrate in order to prevent charging effects. The Cr(C_6_H_6_)_2_ precursor was preheated to 80 °C for at least 30 min. The GIS nozzle with an inner diameter of 0.5 mm was tilted at 30° and positioned 100 µm above the substrate and 100 μm away from the beam center. The Me_3_CpPtMe precursor was preheated to 45 °C, and the GIS was tilted to 60° with the identical positioning. The background pressure was 4 × 10^−7^ mbar and rose to 5 × 10^−7^ mbar for Cr(C_6_H_6_)_2_ and 6 × 10^−6^ mbar for Me_3_CpPtMe during deposition at room temperature. To prevent any mechanical or beam drift, a waiting time of 10 min was introduced right before the start of the deposition process. A series of test depositions with varying beam current (25–6300 pA) and beam size (300–1400 nm) was performed to find optimal conditions in terms of unwanted shape artifacts. Indented deposits were fabricated using 5 keV beam energy at 400 pA beam current and beam size (beam blur setting) of 1400 and 800 nm. Special care was applied with regard to astigmatism correction and aperture alignment, as any aberrations will cause substantial deviations of the deposit shape from a fully symmetric disk-like shape. Depositions were performed at 20 °C substrate temperature and in addition at 10, 15, 30, 35 and 40 °C using a self-made cryo-stage made from copper and equipped with a heater. The stage was cooled via a thick strand of copper wires connected to a Meissner trap cooled with liquid nitrogen. The temperature was controlled via a heating element inserted as an interface layer between the strand and the stage itself. To reach a stable temperature, the substrate was first cooled to 5 °C below the target temperature and then heated up. Profiles of the deposits were obtained using an AFM (Nanosurf EasyScan 2) with an uncertainty of ±3 nm.

## Conclusion

The introduced method enables the determination of the sticking coefficient using indented deposits fabricated by FEBID with a significantly defocused electron beam in the framework of the continuum model. The simplicity and self-consistency of the approach decouples it from error sources typically associated with other parameters needed for the continuum model. The method is primarily useful for the parametrization of a precursor for further usage in a dedicated simulation. The application range of the method is limited for slowly diffusing precursor molecules, such as Me_3_CpPtMe.

The derived sticking coefficient for the Cr(C_6_H_6_)_2_ and Me_3_CpPtMe precursors for our setup are comparable to the value reported for W(CO)_6_, but substantially lower than the estimates previously made for Me_3_CpPtMe and other precursors. This implies that only a tiny fraction of impinging precursor molecules sticks to the surface and contributes to the replenishment of the local coverage.

The observed decrease of the sticking coefficient with increasing temperature for Cr(C_6_H_6_)_2_ together with the consideration of a similar behavior observed for lighter molecules suggests a weak temperature dependence of the sticking coefficient already at room temperature.

## Supporting Information

File 1 Additional supplementary information.

## Data Availability

Data generated and analyzed during this study is available from the corresponding author upon reasonable request.
