# Supplementary material for: Precursor sticking coefficient determination from indented deposits fabricated by electron beam induced deposition
Source: Beilstein J Nanotechnol. 2025 Jan 13;16:35–43. doi: 10.3762/bjnano.16.4 (PMC11744684; doi:10.3762/bjnano.16.4)
Supplement: File 1 — Additional supplementary information. [file Beilstein_J_Nanotechnol-16-35-s001.pdf]

BEILSTEIN JOURNAL OF NANOTECHNOLOGY

# Supporting Information

for

# Precursor sticking coefficient determination from indented deposits fabricated by electron beam induced deposition

Alexander Kuprava and Michael Huth

*Beilstein J. Nanotechnol.* **2025,** *16,* 35–43. doi:10.3762/bjnano.16.4

# Additional supplementary information

License and Terms: This is a supporting information file under the terms of the Creative Commons Attribution License (https://creativecommons.org/licenses/by/4.0). Please note that the reuse, redistribution and reproduction in particular requires that the author(s) and source are credited and that individual graphics may be subject to special legal provisions.
The license is subject to the *Beilstein Journal of Nanotechnology* terms and conditions: (https://www.beilstein-journals.org/bjnano/terms)

# Section 1 – Dissociated molecule fragment volume

The dissociated molecule fragment volume *V* was estimated based on the precursor mass density, deposit composition, and dissociation behavior. The mass density for the discussed precursors is 1.6 g/cm$^3$ for $Cr(C_2H_2)_6$ and 1.88 g/cm$^3$ for $Me_3PtCpMe$. The first step in the calculation of a value range for the fragment volume is based on the assumption that a given precursor molecule may be subject to different degrees of dissociation. The full range of dissociation degrees of a molecule spans from the lowest, when only the loss of one or a few hydrogen atoms takes place, to the full dissociation with only the metal core remaining. Assuming that at the lowest dissociation degree the density of the deposit is equal to the solid precursor density and at the highest degree it is equal to the density of the precursor core metal at normal conditions, the volume value ranges are as follows: $Cr(C_2H_2)_6$: 0.012–0.216 nm$^3$ and $Me_3PtCpMe$: 0.015–0.294 nm$^3$. Now we aim for narrowing this span taking the observed deposition composition into account. The volume of the dissociated fragment is proportional to the amount and size of the ligands remaining after dissociation. The amount can be estimated from the deposit composition by comparing carbon to metal content ratio. From a wide range of data available for $PtC_x$ deposits, it is evident that one to three methyl groups are being cleaved on average, which is supported by studies on $Me_3PtCpMe$ dissociation [1–3]. Assuming that a loss of a methyl group accounts for a 12% ± 5% loss in fragment volume, the maximal volume loss of a dissociated fragment is 36% ± 15%. We take 30% of volume loss based on previous composition measurements and the deposition conditions in our experiments. Hence, for a $PtC_x$ deposit the average dissociated molecule fragment volume is 0.20 ± 0.08

$nm^3$. In the case of $Cr(C_2H_2)_6$, our composition measurements show less than 10 at. % of Cr. We attribute this low Cr content to the high stability of benzene rings. Effectively, molecules are mostly deposited with all ligands intact. To account for the rare event of a benzene ring cleavage, the volume of the fragment is taken as 7% ± 5% smaller than that of the precursor volume. The resulting volume of the dissociated molecule fragment for $Cr(C_2H_2)_6$ is 0.19 ± 0.04 $nm^3$, which is within our estimation approach.

## Section 2 – Surface precursor flux

The estimation of the precursor flux has several error sources. In this work we use a total gas throughput calculation based on the operating (deposition) SEM pressure and pumping speed which then is assumed to be equal to the precursor gas flux at the GIS nozzle exit. To begin with, the pumping speed is estimated from the turbo pump throughput and a factor accounting for throughput reduction due to pipe conductance values, pump age, among other reasons. We assume this factor to be 0.8±0.1. Regarding the SEM operating pressure, our chamber pressure gauge resolution yields measured pressure changes in 3.2e-8 mbar steps. Furthermore, under Cr-precursor flux conditions the measured pressure is hardly different from our SEM base pressure. This introduces a significant error source in the estimation. The precursor flux is calculated from the total gas throughput with the assumption that the precursor gas outflow is significantly higher than that of background gases. This is valid when the operating pressure is at least one order of magnitude higher than the base pressure caused by desorption of the different background gases from inside the vacuum chamber with all its installations. However, in the case of low partial pressure,

such as for the Cr-precursor, the resulting precursor flux is comparable to the background gas outflow, which results in an overestimation of the precursor flux since a large fraction of the total gas flux stems from the background gases. To compensate for this, we assume the Cr-precursor partial pressure to be half as high as the base pressure. In the next step, the total precursor flux is then reduced by a factor corresponding to the spread of the gas jet from the GIS needle in dependence of the proximity of the beam incidence point to the area with the highest local precursor flux. The factor is calculated based on the GIS position and orientation in relation to the surface assuming a molecular gas flow regime. For different angles and beam proximity values the resulting error in the factor varies noticeably, see Figure 7 in [4] for a reference. We allow an uncertainty of 3º of the GIS tilt angle. The resulting precursor flux values with uncertainties are the following: $\Phi(Cr(C_2H_2)_6) = 210 \pm 61$ nm $^{-2}s^{-1}$, $\Phi(Me_3PtCpMe) = 1900 \pm 274$ $nm^{-2}s^{-1}$.

# Section 3 – Diffusive replenishment assessment

In our work, the diffusion contribution at the beam center is neglected completely due to the large beam size and resulting low diffusive replenishment conditions as is described in the paper by Szkudlarek et al. [5]. Assuming for the diffusion coefficient $D$=5·10$^6$ nm$^2$/s, residence time $\tau$=1 ms, an 800 nm wide beam, and precursor supply as derived in the current work, the value of diffusive replenishment according to

$$\tilde{\rho}_{out} = \frac{2\sqrt{D\tau_{out}}}{FWHM_B} \; ; \quad \tau_{out} = \left(\frac{s\Phi}{\mathrm{n}_0} + \frac{1}{\tau}\right)^{-1}$$

is $\tilde{\rho}_{out} = 0.14$, where $FWHM_B$ is the beam width, $n_0$ is the maximum precursor coverage, and $\Phi$ is the surface precursor flux.

## Section 4 – Beam intensity profile

In the Model section of the main text, the growth regimes are discussed under the assumption of a Gaussian beam intensity profile. The beam profile plays an important role in the precursor coverage distribution and mass transport, and the intensity distribution change can affect the resulting growth profile. However, the intensity distribution fluctuations at beam currents suggested for the presented method do not affect the sticking coefficient determination. Under extreme MTL conditions, deviations of the intensity distribution from a Gaussian have a negligible influence on the growth rate. A high current flux condition at the beam center ensures complete consumption of the precursor supplied via adsorption and, as it is discussed in Section 2, mass transport is negligible. The growth rate at the beam center is at its maximum under such extreme MTL conditions. Thus, any fluctuations in beam intensity would not be reflected in the growth rate, the deposit height at the beam center and, thus, the deduced sticking coefficient.

## References

(1) Muthukumar, K.; Jeschke, H. O.; Valentí, R. *Beilstein Journal of Nanotechnology* **2018**, *9*, 711–720.

(2) Engmann, S.; Stano, M.; Matejčík, Š.; Ingólfsson, O. *Physical Chemistry Chemical Physics* **2012**, *14*, 14611–14618.

(3) Wnuk, J. D.; Gorham, J. M.; Rosenberg, S. G.; Van Dorp, W. F.; Madey, T. E.; Hagen, C. W.; Fairbrother, D. H. *Journal of Physical Chemistry C* **2009**, *113*, 2487–2496.

(4) Friedli, V.; Utke, I. *J Phys D Appl Phys* **2009**, *42*, 125305.

(5) Szkudlarek, A.; Szmyt, W.; Kapusta, C.; Utke, I. *Appl Phys A Mater Sci Process* **2014**, *117*, 1715–1726.
